# The Epidemiology of African Swine Fever in “Nonendemic” Regions of Zambia (1989–2015): Implications for Disease Prevention and Control

**DOI:** 10.3390/v9090236

**Published:** 2017-08-23

**Authors:** Edgar Simulundu, Caesar H. Lubaba, Juanita van Heerden, Masahiro Kajihara, Liywalii Mataa, Herman Moses Chambaro, Yona Sinkala, Samuel Munalula Munjita, Hetron Mweemba Munang’andu, King Shimumbo Nalubamba, Kenny Samui, Girja Shanker Pandey, Ayato Takada, Aaron S. Mweene

**Affiliations:** 1 Department of Disease Control, School of Veterinary Medicine, University of Zambia, Lusaka 10101, Zambia; kennysamui@yahoo.com (K.S.); pandeygs@gmail.com (G.S.P.); atakada@czc.hokudai.ac.jp (A.T.); asmweene04@yahoo.com (A.S.M.); 2 Department of Veterinary Services, Ministry of Fisheries and Livestock, Lusaka 10101, Zambia; caesar.lubaba@gmail.com (C.H.L.); lmataa_nmataa@yahoo.com (L.M.); hermcham@gmail.com (H.M.C.); sinkalay@yahoo.co.uk (Y.S.); 3 Agricultural Research Council, Onderstepoort Veterinary Research, Onderstepoort 0110, South Africa; vanheerdenj@arc.agric.za; 4 Division of Global Epidemiology, Hokkaido University Research Center for Zoonosis Control, Sapporo 001-0020, Japan; kajihara@czc.hokudai.ac.jp; 5 Department of Biomedical Sciences, School of Health Sciences, University of Zambia, Lusaka 10101, Zambia; samuelmunjita@gmail.com; 6 Faculty of Veterinary Medicine and Biosciences, Norwegian University of Life Sciences, 0454 Oslo, Norway; hetroney.mweemba.munangandu@nmbu.no; 7 Department of Clinical Studies, School of Veterinary Medicine, University of Zambia, Lusaka 10101, Zambia; king.nalubamba@unza.zm; 8 Global Institution for Collaborative Research and Education, Hokkaido University, Sapporo 001-0020, Japan

**Keywords:** *Asfarviridae*, African swine fever, domestic pigs, phylogenetic analysis, molecular epidemiology, Zambia

## Abstract

African swine fever (ASF) is a highly contagious and deadly viral hemorrhagic disease of swine. In Zambia, ASF was first reported in 1912 in Eastern Province and is currently believed to be endemic in that province only. Strict quarantine measures implemented at the Luangwa River Bridge, the only surface outlet from Eastern Province, appeared to be successful in restricting the disease. However, in 1989, an outbreak occurred for the first time outside the endemic province. Sporadic outbreaks have since occurred almost throughout the country. These events have brought into acute focus our limited understanding of the epidemiology of ASF in Zambia. Here, we review the epidemiology of the disease in areas considered nonendemic from 1989 to 2015. Comprehensive sequence analysis conducted on genetic data of ASF viruses (ASFVs) detected in domestic pigs revealed that *p72* genotypes I, II, VIII and XIV have been involved in causing ASF outbreaks in swine during the study period. With the exception of the 1989 outbreak, we found no concrete evidence of dissemination of ASFVs from Eastern Province to other parts of the country. Our analyses revealed a complex epidemiology of the disease with a possibility of sylvatic cycle involvement. Trade and/or movement of pigs and their products, both within and across international borders, appear to have been the major factor in ASFV dissemination. Since ASFVs with the potential to cause countrywide and possibly regional outbreaks, could emerge from “nonendemic regions”, the current ASF control policy in Zambia requires a dramatic shift to ensure a more sustainable pig industry.

## 1. Introduction

African swine fever (ASF) is an acute, highly contagious and deadly disease of domestic pigs, wild boars and other members of the family *Suidae*. ASF virus (ASFV), a large and complex DNA arbovirus, which is currently the only member of the family *Asfarviridae*, genus *Asfivirus* [1], is the causative agent of this disease. Depending on the viral isolate, the ASFV genome varies in length from about 170 to 193 kilobase pairs [2,3].

ASF was first observed in Kenya in 1909 following the introduction of European (exotic) domestic pigs [4]. Currently, the disease is endemic in many countries in sub-Saharan Africa, where the virus is maintained in a sylvatic cycle and/or a domestic pig cycle [5,6]. Typically, sylvatic cycle maintenance of ASFV involves asymptomatic infection of wild pigs (particularly warthogs) and soft ticks of the genus *Ornithodoros*. On the other hand, ASFV can be maintained in domestic pigs via a pig–tick cycle, which does not involve warthogs and a pig–pig cycle through direct contact between infected and susceptible animals. Whilst the acute form of the disease is frequently observed in domestic pigs, other clinical manifestations such as chronic and subclinical or unapparent which may be associated with moderate or low virulent strains have been reported in endemic regions [7].

Due to its high mortality rates of about 95–100% in peracute and acutely infected pigs, coupled with lack of vaccines or effective drugs for prevention or treatment, ASF is considered the most important contagious disease threatening the development of the pig industry in Africa. While the disease can devastate both the commercial and subsistence pig production sectors, the rural smallholder pig farmers are usually the most affected [8]. In the absence of compensation schemes to mitigate the socioeconomic impact of ASF, smallholder pig farmers are often left with no or limited resources to restart production.

ASF has not only plagued sub-Saharan countries. Outside Africa, ASF was first introduced into Europe (Portugal) in 1957 and subsequently spread within the Iberian Peninsula and other European countries [8]. The disease also affected several countries in the Americas during the early 1970s up to about 1981. With the exception of the island of Sardinia (Italy), ASF was successfully eradicated in all these regions. However, in 2007, ASF spread from southeastern Africa to the Republic of Georgia and throughout the Caucasus region, the Russian Federation and was subsequently introduced into some countries of the European Union [9,10,11,12]. The presence of ASFV in both wild boars and domestic pigs in these regions poses a constant threat to the European Union swine sector and has heightened the risk to the global pig industry.

In Zambia, ASF was first reported in 1912 near Fort Jameson, now Chipata District, in Eastern Province [13]. With the official recognition of the endemicity of ASF in native (free-roaming) pigs in that province in 1965, coupled with the absence of reports of the disease in other parts of the country, it was generally believed that only Eastern Province is affected with this disease [13]. Consequently, as a way of controlling the disease from spreading to other parts of the country, a longstanding ban on the export of pigs and their products from Eastern Province to the rest of the country has been in effect since 1965. Moreover, strict quarantine measures were implemented at the Luangwa River Bridge, the only surface outlet from the Province. Despite all these measures, for the first time in 1989, an ASF outbreak occurred in Kabwe, Central Province, located hundreds of kilometers outside the endemic province [14,15]. Since 1989, sporadic ASF outbreaks have occurred in almost all the provinces of the country. While outbreaks of ASF in areas considered to be nonendemic might highlight our limited understanding of the transmission dynamics and epidemiology of this disease, they also question the effectiveness of some of the current disease preventive measures applied in Zambia. Moreover, the numerous outbreaks in these areas bring into question the relevance of stringent control measures, particularly the long-standing ban on the movement of pigs and pig products out of Eastern Province to other parts of the country.

In the recent past, molecular characterization of distinct genome regions of the ASFV has proved to be very useful in elucidating the origin and transmission pathways of ASF during outbreak scenarios [9,10,11,16,17,18,19,20]. The current practice of studying the molecular epidemiology of ASF is through sequence analysis of the 3′-end of the *B646L* gene encoding the p72 protein [21], which differentiates at least 23 genotypes [22,23,24]. Full-length sequence analysis of the *E183L* gene, which encodes the envelope protein p54, has been shown to provide intermediate levels of virus discrimination in placing isolates into major subgroups [25]. The *p54* phylogeny generally follows the *p72* pattern. Enhanced intra-genotypic discrimination is achieved through sequence analysis of the central variable region (CVR) within the *B602L* gene [25,26]. Furthermore, analysis of other genome regions such as *CP204L* gene (coding the phosphoprotein p30) and the region located between the *I73R* and *I329L* genes, could be used for discrimination between closely related ASFVs [9,11].

The present study reviewed the history of ASF epidemics and investigated the molecular epidemiology of the disease in regions currently considered nonendemic in Zambia. The possible measures that could be explored for disease prevention and control are discussed.

## 2. History of ASF Outbreaks in Nonendemic Areas outside Eastern Province

The history of ASF epidemics in nonendemic areas was studied by collating and analyzing data from published articles as well as various outbreak reports submitted to bodies such as the World Organisation for Animal Health (OIE) or the Program for Monitoring Emerging Diseases (ProMED).

### 2.1. ASF Outbreak in Kabwe, Central Province (1989)

**Figure 1:**
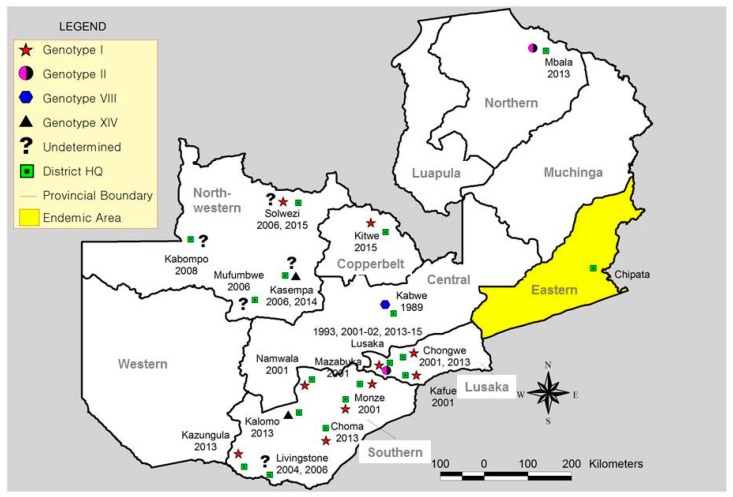
Map showing dates of African swine fever (ASF) outbreaks and the spatial distribution of *p72* African swine fever virus (ASFV) genotypes identified in “non-endemic” areas in Zambia (1989–2015).

The first outbreak of ASF outside the endemic province (i.e., Eastern Province), occurred at Mpima Seminary in Kabwe District, Central Province, in May 1989 [14,15] (Figure 1). The affected piggery had 164 pigs. On 6 May 1989, one pig within the parental stock suddenly showed signs of illness and died the following day. By 23 May, when stamping-out control measures were implemented, 30 pigs had died. Laboratory confirmation was through direct and indirect immunofluorescent antibody tests on tissues (lymph node, lung, spleen) and serum samples, respectively, which were performed at Central Veterinary Laboratory in Malawi and at Pirbright Institute in the UK.

Epidemiological investigations provided no evidence of a local origin of the ASFV, which might have been responsible for the outbreak as there were no sylvatic hosts (soft ticks and warthogs) on the premises and surrounding areas, leading to the suspicion that the feeding of pigs with contaminated kitchen remains could have contributed to this outbreak [15].

### 2.2. ASF Outbreaks in Lusaka Province (1993)

In 1993, an outbreak of ASF, affecting several farms, was reported in the Eastern part of Lusaka District [27]. This outbreak, which placed more than 10,000 pigs at risk, was effectively controlled through strict quarantine, restriction of movement of pigs, closure of slaughterhouses and ban on import of pigs and pig products as well as stamping out. Although the origin of the ASFV associated with this outbreak was not determined, illegal movement of pigs from an infected area (probably Eastern Province) was suspected to have triggered the outbreaks. 

### 2.3. ASF Outbreaks in Lusaka and Southern Provinces (2001–2002)

During 2001–2002, Zambia reported 12 ASF outbreaks in domestic pigs to the OIE that affected Namwala, Monze, Mazabuka, Kafue, Lusaka and Chongwe Districts [22,28,29] (Table S1 and Figure 1). In Namwala District, suspected ASF cases were first reported in March 2001 and appear to have started at Nanzhila near Bilili game management area (GMA), adjacent to the Kafue National Park (KNP). Immediately after the outbreak occurred, farmers started selling their pigs to traders to avoid total losses. The traders transported the pigs to other districts, particularly Kafue and Lusaka Districts. In Mazabuka District, the first cases of ASF were observed during the first week of August 2001 at a farm located approximately 100 m from a livestock loading bay on the Livingstone-Lusaka highway. The disease then spread to several villages. Ironically, although the farmers initially suspected malicious poisoning as the cause of disease, the disease appears to have spread through movement of pork from household to household. During this period, ASF also affected several smallholder farmers in Monze District. It was epidemiologically worth noting that among the affected farmers in Monze was one who bought pigs and reared them at home prior to transporting them to Chibolya market in Lusaka District for sale. Chibolya market possesses a slaughter slab where animals particularly from smallholder farmers in rural areas are slaughtered [30].

In Kafue District, ASF occurred in August/September 2001 in two high-density residential areas, namely Zambia and Soloboni compounds. Epidemiological investigations conducted by government officials indicated that pig traders were buying animals from Southern Province (e.g., Mazabuka District) and were transporting them to Kafue District en route to Chibolya market. In Lusaka District, ASF affected mainly Lusaka North, Lusaka West, Makeni, and Lusaka East areas. In Lusaka North, suspected ASF cases were observed during the first week of August 2001 on two commercial properties with a total susceptible pig population of 1105 [31]. At least 431 pigs were reported to have died from the disease. Epidemiologically, it was suspected that the source of infection may have been a neighboring abattoir and that the disease could have spread through illegal movement of infected pigs from Southern Province. In Lusaka West and Makeni area, suspected cases of ASF occurred at farms with a history of purchasing pigs from Chibolya market. Similarly, the outbreak in Chongwe District occurred at a single smallholder farm whose owner had purchased a sow at Chibolya market.

On 10 September 2002, an outbreak of ASF occurred at a commercial property in Lusaka East [32]. The establishment had a processing plant, for both beef and pork within the farm, which was located a few meters away from the piggery. Epidemiological investigations by government officials revealed that high pig mortalities were first observed in July/August 2002 after the farmer had brought pigs and carcasses that were purchased in Monze District, which was under quarantine at the time due to occurrence of ASF outbreaks. To control the outbreak, the Zambian Veterinary department destroyed at least 1100 pigs on the farm on 21 September 2002 [32].

### 2.4. ASF Outbreaks in Livingstone, Southern Province (2004 and 2006)

In April 2004, a suspected outbreak of ASF was reported from Livingstone District for the first time [33]. The outbreak affected a household with a pig herd of 21 reared in a backyard pen. The owner first noticed three pigs showing anorexia and general weakness on 16 April 2004 and the following day two more pigs showed clinical signs. The pigs did not respond to penicillin treatment and two died on 19 April 2004. The clinical signs observed were highly suggestive of ASF. Further investigations revealed that about 280 pigs had died at a commercial farm situated approximately 7 km South East of Livingstone town around early March, which was not reported to the Zambian Veterinary department. 

In January 2006, ASF affected two farms in Livingstone District [34]. A total of 145 exotic pigs (large white and landrace) reared in a semi-intensive system either died from the infection or were stamped out during implementation of control measures. It was speculated that ASFV-contaminated wild swine meat, probably poached from Livingstone Zoological Park might have been the source of the infection [34]. Another possible source of the infection may have been through fomites (vehicles, feed, etc.) or swill feeding.

### 2.5. ASF Outbreaks in Northwestern Province (2006–2008)

**Figure 2:**
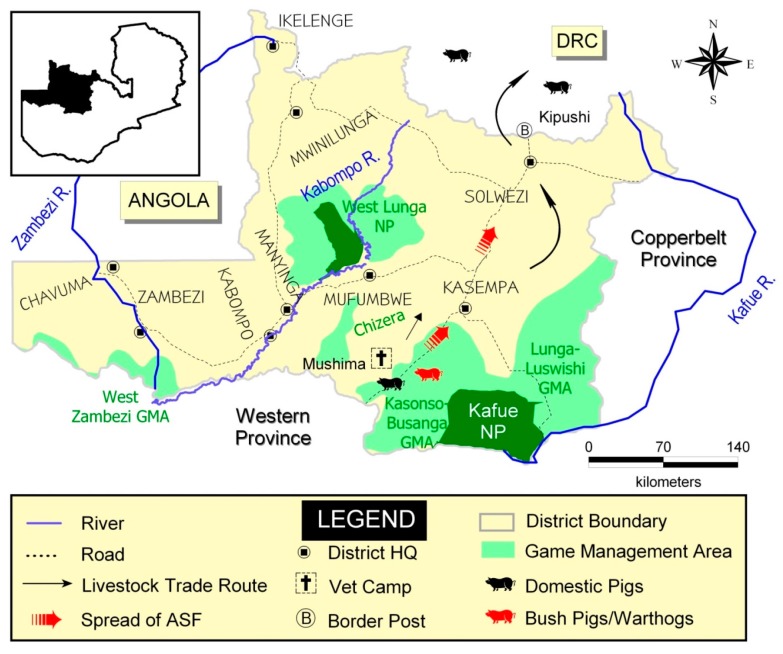
Map of Northwestern Province showing the spread of ASF along livestock trade route during the outbreaks of 2006–2008.

ASF had not been recorded in Northwestern Province until 2006 when unofficial reports indicated that a number of domestic pigs in Mufumbwe District showed clinical signs resembling those of ASF. This was in an area situated in the Kasonso-Busanga GMA near KNP. ASF was officially diagnosed in the province in 2007 following disease outbreaks, which affected four districts, namely Mufumbwe, Kasempa, Kabompo and Solwezi [35]. The outbreaks started on 1 November 2007. At the time they were declared resolved, the province recorded 151 cases with 147 deaths [36]. Moreover, it was reported in 2008 that at least 3000 swine had died of the infectious disease in the province [37]. The source of the outbreaks or origin of infection was thought to be contact with wild swine and/or illegal movement of pigs. Noteworthy is the observation that the spread of ASF in the province appeared to follow the major livestock trade route from Mufumbwe through Kasempa and Solwezi to the Democratic Republic of Congo (DRC) via Kipushi border post (Figure 2).

### 2.6. ASF Outbreaks in Zambia during 2013–2015

During 2013–2015, Zambia experienced unprecedented widespread outbreaks of ASF in domestic pigs that involved six provinces and ten districts [16,38,39] (Figure 1). In April 2013, ASF outbreaks were reported in Mbala District (Northern Province) among domestic pigs reared in villages along the border with neighboring Tanzania. In July, the same year, ASF outbreaks were reported in Kazungula District of Southern Province. Before the end of 2013, outbreaks occurred in several other districts including Kalomo, Choma, Lusaka and Chongwe (Figure 1). These outbreaks appeared to follow the major trade route along the Livingstone-Lusaka highway. In Lusaka Province, a high-density residential area (Linda compound in Chilanga) was affected [39]. By the year 2015, ASF outbreaks were reported in Kitwe, Solwezi and Kasempa Districts. Genetic characterization showed the involvement of multiple genotypes of ASFVs, particularly genotypes I, II and XIV [16] (Figure 1). Among these, genotype I ASFV spread more widely through trade and movement of pigs. While genotype I and XIV ASFVs emerged from Southern Province, genetically distinct genotype II viruses were detected in Mbala (Northern Province) and Chipata Districts (Eastern Province) [16]. Whereas genotype II ASFV, which was found in Mbala District (Georgia 2007/1-like virus), appears to have been introduced from neighboring Tanzania through livestock trade [16], the Chipata virus appears to have emerged locally. Overall, epidemiological investigations of the 2013–2015 ASF outbreaks in Zambia revealed that trade and movement of pigs and/or their products facilitated the widespread dissemination of ASFV, with Southern Province being a major source of viruses responsible for the outbreaks [16].

## 3. Brief History and Status of ASF in Neighboring Countries of Zambia

To better understand the epidemiology of ASF in Zambia, a brief history and status of the disease in the surrounding countries is warranted, as this may help in the identification of disease trends, possible origin of ASFVs and transmission dynamics in the region. Zambia is a landlocked country surrounded by eight countries; namely Angola, Botswana, DRC, Malawi, Mozambique, Namibia, Tanzania and Zimbabwe. In Angola, ASF was first reported in 1933 and is likely to be endemic in domestic pigs and is present in warthogs [40,41,42]. ASF outbreaks in Botswana are limited and rare, with the latest outbreak being reported in 1999 [23]. The outbreaks are usually associated with contact with warthogs. While there is lack of published data on the history of ASF in DRC, recent reports suggest that the disease situation in this country could be dire. For instance, in 2011, DRC reported 84 outbreaks, which led to a loss of 105,614 pigs [43]. Genetic assessment of ASFVs associated with outbreaks during 2005 to 2012 in DRC revealed the co-circulation of multiple *p72* genotypes (I, IX and XIV) with possible transboundary movements of viruses involving neighboring countries such as Uganda, Zambia and Brazzaville, Republic of Congo [44]. In Malawi and Mozambique, ASF was confirmed during early 1960s [45,46,47] and is endemic in domestic pigs in both countries [42]. Studies conducted in the southwestern part of Malawi, bordering on Mozambique and Zambia, have demonstrated the existence of a transmission cycle involving domestic pigs and ticks of the *Ornithodoros* genus inhabiting pig shelters [48]. Prior to 1994, ASF in Mozambique was restricted to an area north of the Save River [49]. However, following the 1994 major outbreak at the Veterinary Faculty in Maputo, the geographical distribution of ASF in Mozambique changed dramatically as sporadic outbreaks, mostly associated with movements of pigs and their products occur across the country [49]. Recently, the possible involvement of a sylvatic cycle in the epidemiology of ASF in Mozambique was demonstrated [50]. The ASF situation in Namibia is similar to that in Botswana and Zimbabwe, characterized by occasional outbreaks which are associated with contact with warthogs. Namibia experienced outbreaks in 2009 in the Omusati region along the border with Angola [51]. Until July 2015 when an ASF outbreak was identified in Mashonaland Central Province along the border with Mozambique, Zimbabwe had no reported outbreaks since 1992 [52]. ASFV genotype II was associated with the outbreak, suggesting that this viral genotype may be spreading within the southeastern African region [52]. Tanzania has experienced an increase in the number of ASF outbreaks in different parts of the country since the late 1990s, which mostly have been associated with illegal movement of pigs and their products as well as swill feeding [17,18,53,54].

## 4. Genetic Analysis of ASFVs Detected in Zambia

Molecular characterization of previously sequenced genetic data (found in the GenBank) of Zambian ASFVs and those sequenced in this study was performed following standard protocols as recommended by OIE [55]. Specifically, we conducted genetic analyses of nucleotide and/or amino acid sequences of the *p72*, *p54*, *p30* and the CVR. We sequenced the *p30* gene of ten ASFVs that were associated with recent outbreaks (2013–2015) and the *CVR* gene of 17 ASFVs from Zambian samples archived at the OIE ASF reference laboratory at Onderstepoort Veterinary Research, Agricultural Research Council, South Africa. The *p30* gene was amplified and sequenced using the primer pair p30-F (5′-ATGAAAATGGAGGTCATCTTCAAAAC-3′) and p30-R (5’-AAGTTTAATGACCATGAGTCTTACC-3′) [9]. Sequencing of the CVR was conducted using the primer pair ORF9L-F/ORF9L-R [25] and/or CVR-1 (5′-ACTTTGAAACAGGAAAC(AT)AATGATG-3′) and CVR-2 (5’-ATATTTTGTAATATGTGGGCTGCTG-3′). DNA purification from agarose gels, sequencing, assembly and editing of sequences was performed as previously described [16]. The sequences from this study were deposited in the GenBank under accession no. LC213609-LC213618, MF322710-MF322716, MF322718-MF322724, MF359235, MF359237, and MF359239. Phylogenetic analysis was performed using the neighbor-joining method, with evolutionary distances being calculated using the *p*-distance approach. The phylogenetic trees were generated using MEGA6 software, version 6.06 [56].

### 4.1. Phylogenetic Analysis of the p72 (B646L) Gene

**Figure 3:**
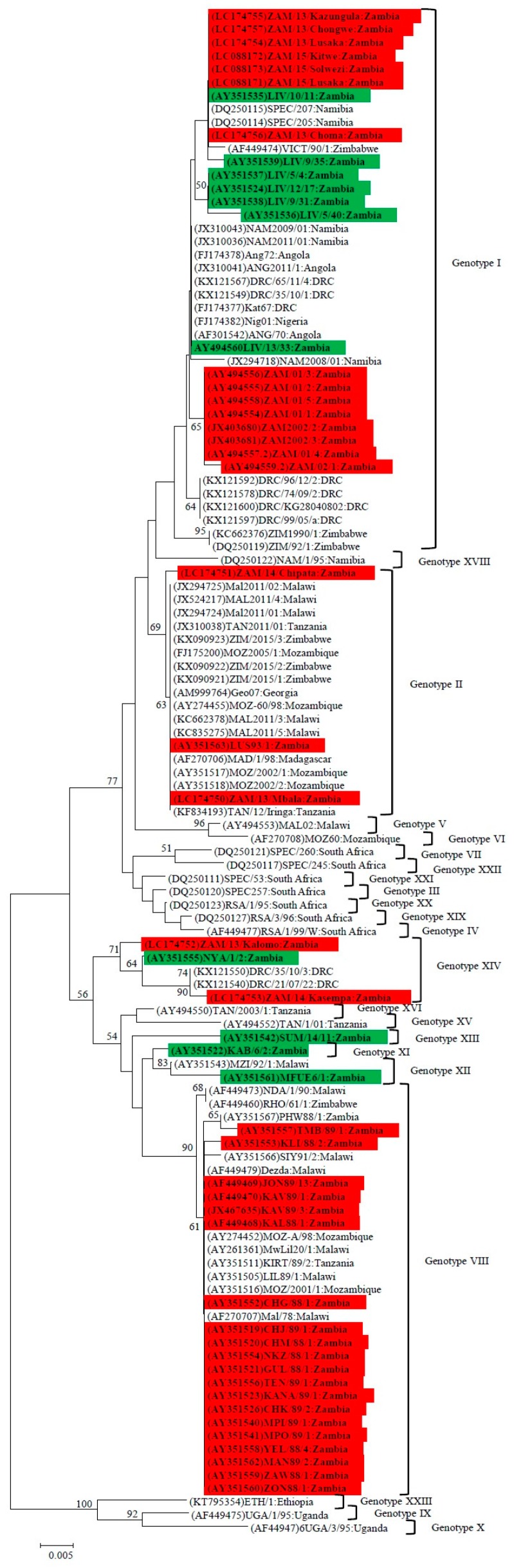
Phylogenetic relationships of nucleotide sequences of *p72* gene of ASFVs detected in pigs and soft ticks in Zambia. The analysis was based on 403 bp of the *p72* gene. The evolutionary history was inferred using the minimum evolution (ME) method with evolutionary distances being computed using the p-distance method. The ME tree was searched using the close-neighbor-interchange (CNI) algorithm at a search level of 1. The neighbor-joining algorithm was used to generate the initial tree. Numbers at branch nodes indicate bootstrap values ≥50%. The GenBank accession no. of strains included in the analyses are indicated in parenthesis. Virus strains characterized in the present study detected in domestic pigs are shaded in red while those from ticks are shaded in green. Bar, number of substitutions per site.

Phylogenetic analysis of the *p72* gene of ASFVs detected in domestic pigs and soft ticks in Zambia confirmed the identification of at least seven genotypes in the country, namely I, II, VIII, XI, XII, XIII and XIV [16,22].(Table S1 and Figure 3). Of these, four genotypes (i.e., I, II, VIII and XIV) were involved in causing outbreaks in areas outside Eastern Province of Zambia. Whilst genotype I ASFVs that were associated with outbreaks in Lusaka and Southern Province during 2001–2002 formed a distinguishable cluster, viruses that were detected in domestic pigs during 2013–2015 outbreaks showed close relationships with those from ticks in Livingstone Game Park (Southern Province) as well as pig-associated viruses in neighboring countries such as Namibia, Angola and DRC (Figure 3).

Another large cluster of Zambian viruses consisted mostly of genotype VIII ASFVs, which were isolated from domestic pigs in Eastern Province (Figure 3). These viruses have been involved in many outbreaks in several other countries including Malawi, Mozambique, Tanzania and Zimbabwe [22] (Figure 3).The first ASF outbreak outside the endemic zone in Zambia, which occurred at Mpima Seminary in 1989, was caused by a genotype VIII ASFV (MPI 89/1, accession no. AY351540) (Figure 3). Genotype II viruses detected in Lusaka in 1993 (LUS 93/1 accession no. AY351563) and in Mbala in 2013 (ZAM/13/Mbala accession no. LC174750) were highly similar to the Georgia 2007/1 isolate. However, the virus found in Chipata (Eastern Province) was phylogenetically distinct from Georgia 2007/1 (Figure 3). In Southeastern Africa, the spatial distribution of genotype II ASFV has been increasing as it has now been found in seven countries (i.e., Madagascar, Malawi, Mauritius, Mozambique, Tanzania, Zambia and Zimbabwe [8,52] (Figure 3).

Genotype XIV ASFV detected in domestic pigs in Northwestern Province in 2014 grouped with viruses that caused outbreaks in DRC [44] and were phylogenetically distinguishable from those detected in Southern Province of Zambia (Figure 3). It is worth noting that outside Zambia, genotype XIV viruses have only been described in DRC.

Overall, phylogenetic analysis of the *p72* gene of ASFVs in Zambia has revealed that both genotypes I and XIV viruses have been detected in domestic pigs and sylvatic hosts (soft ticks) with the former being found in several other neighboring countries than the later. On the other hand, whilst both genotype II and VIII viruses were also detected in multiple countries, they were predominantly associated with domestic pigs.

### 4.2. Phylogenetic Analysis of the p54 (E183L) Gene

**Figure 4:**
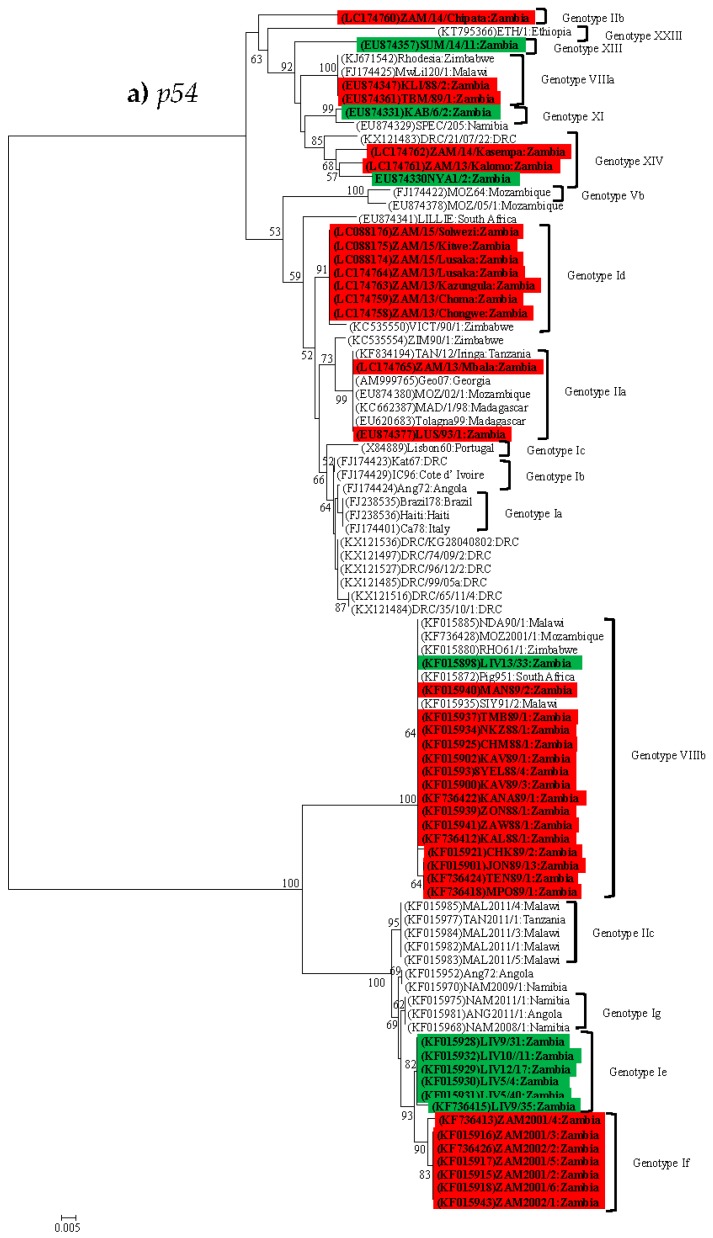

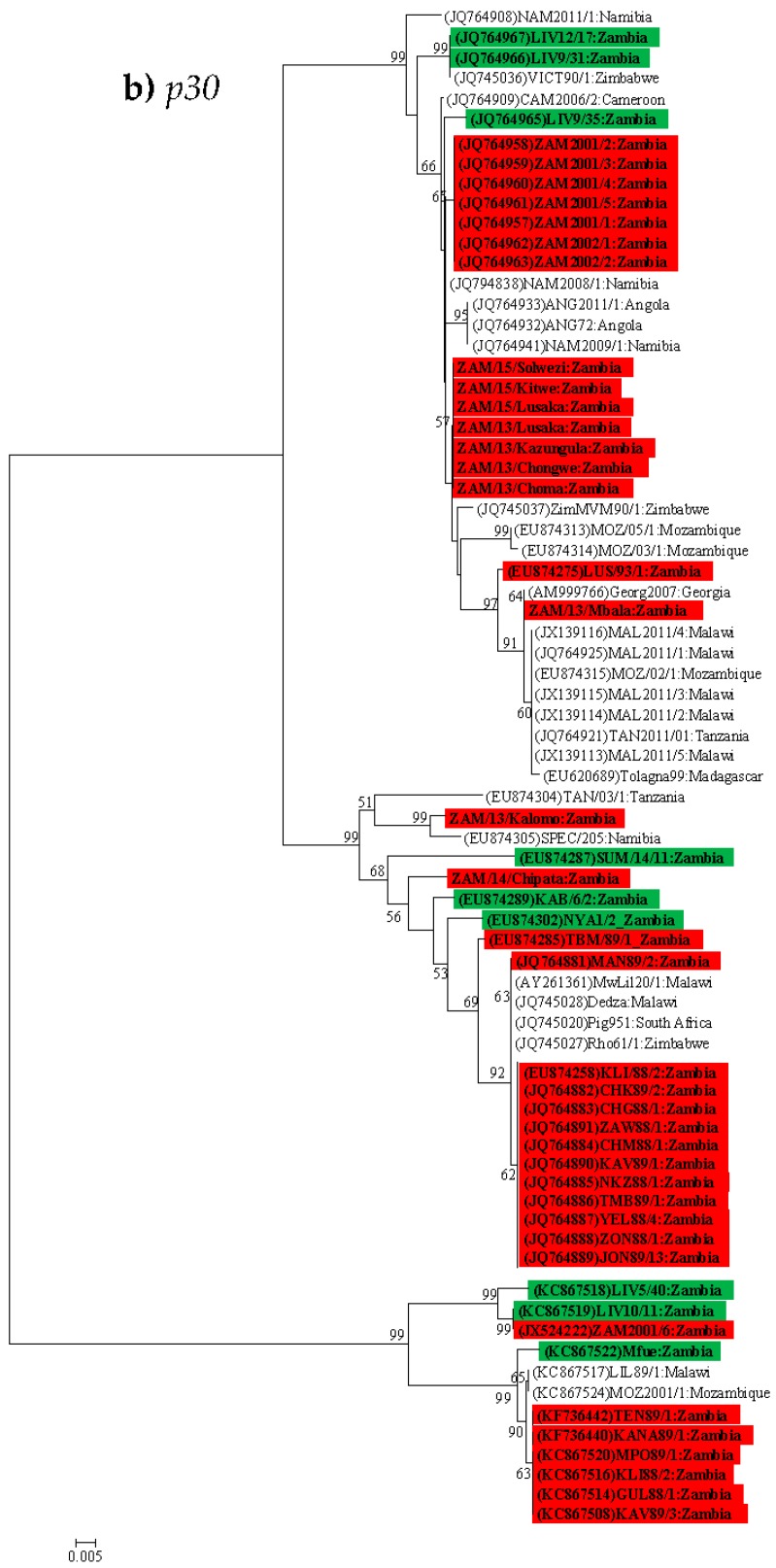
Phylogenetic relationships of the *p54* (**a**) and *p30* (**b**) genes of ASFVs detected in pigs and soft ticks in Zambia. Analysis was based on 483 bp of the *p54* and 528 bp of the *p30* genes. The evolutionary history was inferred using the Minimum Evolution method with evolutionary distances being computed using the p-distance method. The ME tree was searched using the close-neighbor-interchange (CNI) algorithm at a search level of 1. The neighbor-joining algorithm was used to generate the initial tree. Numbers at branch nodes indicate bootstrap values ≥50%. The GenBank accession no. of strains included in the analysis are indicated in parenthesis. Virus strains characterized in the present study detected in domestic pigs are shaded in red while those from ticks are shaded in green. Bar, number of substitutions per site.

In the *p54* genetic tree, whereas *p72* genotype I ASFVs, which were responsible for outbreaks during 2013–2015 belonged to *p54* genotype Id [16,39] together with a virus which was isolated from a tick in Zimbabwe (VICT/90/1, accession no. KC535550), those that were detected in ticks in Livingstone clustered in a different branch and have been assigned to a new *p54* genotype Ie (Figure 4a). Moreover, ASFVs detected in domestic pigs during 2001–2002 clustered together under a new *p54* genotype If. Also, ASFVs detected in Namibia and Angola in 2011 were assigned a new *p54* genotype Ig (Figure 4a). The *p72* genotype II viruses from Zambia separated into two distantly related *p54* genotypes, IIa (Georgia 2007/1-like) and IIb (ZAM/14/Chipata-like) [16] (Figure 4a). Interestingly, some *p72* genotype II viruses detected in Malawi and Tanzania in 2011 clustered in a well-supported (bootstrap value 95%) distinct branch and thus were assigned a novel *p54* genotype IIc (Figure 4a). These findings suggest that genotype II viruses could be more diverse than previously thought. Similarly, *p72* genotype VIII viruses were reclassified into new distantly related *p54* genotypes VIIIa and VIIIb (Figure 4a). The *p54* genotype VIIIa viruses consisted of strains from Malawi, Zambia and Zimbabwe while genotype VIIIb also included viruses from Mozambique and South Africa. Unexpectedly, a *p72* genotype I virus (LIV 13/33 accession no. KF015898) isolated from ticks in Livingstone in 1983 [22] clustered with *p54* genotype VIIIb (Figure 4a). Meanwhile, phylogenetic analysis results for the *p54* gene showed that a *p72* genotype I virus found in Namibia (SPEC/205, accession no. EU874329) clustered with a genotype XI virus (KAB/6/2, accession no. EU874331), which was isolated from a tick in Zambia (Figure 4a). The clustering pattern of genotype XIV ASFVs from Zambia was consistent with that of *p72* (Figure 3 and Figure 4a). Taken together, whilst phylogenetic analysis results of the *p54* gene have demonstrated the utility of this genome region in discrimination of closely related viruses, they have also shown the existence of ASFVs which defy the idea that *p54* phylogeny follows the *p72* clustering pattern.

### 4.3. Phylogenetic Analysis of the p30 (CP204L) Gene

In the *p30* genetic tree, with the exception of one virus (ZAM 2001/6, accession no. JX524222), all *p72* genotype I ASFVs, which were associated with the 2001–2002 and 2013–2015 outbreaks belonged to the same clade (Figure 4b). The *p72* genotype I viruses of 2001–2002 were closely related to an isolate (NAM 2008/1, accession no. JQ794838), which was associated with an ASF outbreak in Namibia in 2008 [57]. This clade included some of the tick-associated ASFVs that belonged to the newly designated *p54* genotype Ie (Figure 4a,b). The other two *p54* genotype Ie strains belonged to a different branch which showed a sister-like relationship to *p72* genotype VIII viruses (i.e., the group represented by Mfue, accession no. KC867522) (Figure 4b). The two Zambian ASFVs (i.e., LUS 93/1 and ZAM/13/Mbala), which were indistinguishable in the *p72* and *p54* genetic trees were genetically differentiated in the *p30* phylogeny (Figure 4b).

The *p72* genotype VIII viruses separated into two groups. The majority of the viruses belonged to a group, which was closely related to an ASFV (MwLil 20/1, accession no. AY261361) isolated in Malawi in 1983. The other group of genotype VIII viruses shared a common ancestor with the Mfue isolate (Figure 4b). Inconsistent with the *p72* and *p54* phylogenetic trees, the *p72* genotype XIV viruses showed a different clustering pattern, as they did not group together (Figure 4b).

### 4.4. Sequence Analysis of the CVR within the B602L Gene

**Figure 5:**
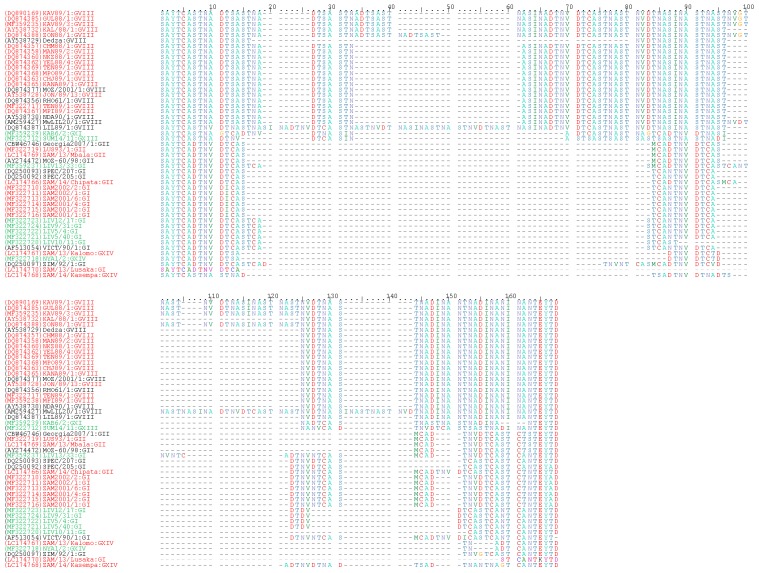
Multiple sequence alignment of amino acids of the tetrameric tandem repeats of the central variable region (CVR) of ASFVs detected in Zambia. The ASFVs characterized in this study are shown in red and green text for viruses detected in domestic pigs and soft ticks, respectively. The GenBank accession or protein identification no. of strains included in the analyses are indicated in parenthesis while the *p72* genotypes are shown after the colon.

Multiple sequence alignment of amino acid sequences of the tetrameric repeats of the CVR of the *B602L* gene identified in Zambia revealed the existence of at least 14 CVR variants (Figure 5 and Table 1). Three variants (represented by CHM 88/1, ZON 88/1 and KAV 89/1) [22] were identified among the Zambian *p72* genotype VIII viruses (Figure 5 and Table 1). With the exception of the *p72* genotype VIII CVR variant represented by ZON 88/1, which was only identified in Zambia, the other two variants have been identified in Malawi and Mozambique [22]. Moreover, by utilizing a combined *p72*-CVR approach, the highest number of genotype VIII virus variants has been detected in Malawi and this provoked the idea that this country could be a possible reservoir/source of these viruses [22].

Five genotype I ASFV CVR variants were identified in this study (Figure 5 and Table 1). Of these, two (represented by ZAM/13/Lusaka: BNAF and ZAM 2002/2: BNAFNBTDBNAF) were detected in ASFVs associated with outbreaks in domestic pigs while the rest were obtained from *Ornithodoros* ticks (i.e., those represented by LIV 13/33: BNADBNAFTBTDBNAF; LIV 12/17: BNAAFNBTAFF; and LIV 10/11: BNAAAAAF). The two tetramer types detected in *p72* genotype I strains from domestic pigs in Zambia were distinct from that identified from outbreaks in southern Africa during 1973–1999 [23] and the thirteen variants recently reported in DRC [42]. 

The two CVR variants identified among genotype II viruses from Zambia included the ASFV found in Mbala, which showed 100% sequence identity to that of the Georgia 2007/1 virus (Figure 5 and Table 1). The other genotype II CVR variant was that of the ASFV identified in Chipata. The two genotype XIV CVR variants identified in this study (ZAM/13/Kalomo: BNWNBVF and ZAM/14/Kasempa: AVVWNVWNVWVVF) were distinct from those characterized in DRC [42] (drc35/10/3: AAAAAAAAAAAAAAAAAAAAAAAAAAAAABNABNBTDBNAAAAAAAAAAAF and drc21/07/22: AVVOVAVVNBVOV). However, the CVR sequence of ZAM/13/Kalomo was 100% identical to that of NYA 1/2 virus which was isolated from a tick in the same district in 1986 (Figure 5 and Table 1). This finding may suggest a possible sylvatic cycle involvement in ASF outbreaks associated with genotype XIV viruses in Zambia. The remaining two CVR variants found in Zambia were for genotypes XI and XIII (Table 1), which were detected in soft ticks and have not been associated with domestic pigs to date.

## 5. Discussion

Molecular epidemiological findings of the current study suggest that with the exception of the Mpima Seminary outbreak of 1989 in Kabwe, the majority of ASF outbreaks in areas currently being considered nonendemic originated outside Eastern Province. While this might imply that the stringent preventive measures prohibiting the export of pigs and pork products from Eastern Province may have been quite effective in limiting the introduction of ASFVs to other parts of the country, it might also indicate a knowledge gap in our understanding of the epidemiology of ASF in Zambia. The idea that illegal transportation of pork products, which might have been fed to pigs and thus triggering the outbreak in Kabwe seems to gain support from our genetic analyses, which showed that the virus (MPI 89/1) was very similar in the *p72* and CVR of the *B602L* genes to ASFVs (e.g., CHJ 89/1 and CHM 88/1) which caused outbreaks in Petauke District in Eastern Province during 1988–1989 (Table S1, and Figure 3 and Figure 5). Therefore, we suggest that the ban on the movement of pigs and their products from Eastern Province be maintained.

The identification of ASFVs, which did not follow the *p72* phylogenetic clustering pattern in the *p54* genetic tree, suggests that such strains could be more prevalent than previously thought. It was interesting to note that whereas *p54* genotype Ia to Id clustered together, they were distantly related to genotypes Ie, If and Ig (Figure 4a). This was true for *p54* genotypes IIa, IIb and IIc as well as for genotypes VIIIa and VIIIb. These observations underscore the need to improve the current ASFV genotyping system, especially for strains whose *p54* phylogeny does not follow that of the *p72* pattern.

Phylogenetic analysis of the *p30* gene sequences of Zambian ASFVs revealed that this genome region could discriminate among closely related viruses. These findings are in contrast to those of a recent study in which *p30* sequences obtained from ASFV collected in Sardinia over a 13-year period showed a remarkable genetic stability and thus could not achieve a finer level of discrimination among closely related viruses using this genome region [58]. However, when combined with analyses of the CVR which revealed the existence of at least 14 variants in Zambia, our genetic analysis findings support the idea of examining several genome regions of ASFVs in order to better understand the epidemiology of ASF.

During the study period, Zambia appears to have suffered at least three prolonged ASF outbreaks, which occurred in 2001–2002, 2006–2008, and 2013–2015. While the lack of genetic data for the 2006–2008 outbreaks in Northwestern Province hindered the unraveling of the molecular epidemiology of ASFVs involved in these epidemics, analysis of epidemiological data showed some similarities between the 2001–2002 and 2013–2015 outbreaks. These outbreaks mostly affected districts located along the line of rail or the Livingstone-Lusaka highway. In both periods, genotype I viruses, which spread to at least two provinces were involved. Although they were phylogenetically distinct, they all appeared to have emerged from Southern Province, where trade and illegal movements of pigs and/or pork products facilitated their spread to multiple districts and provinces. In both occasions, the outbreaks affected high-density residential areas. It is probable that ASF could be endemic in some parts of Southern and/or Northwestern provinces, particularly in areas situated at the wildlife-domestic interface around the KNP. Conducting epidemiological investigations similar to those of the recent study carried out in Mozambique, which provided evidence of the existence of a sylvatic cycle at the wildlife-domestic interface of the Gorongosa National Park [45], could help confirm this possibility. A number of interface areas surround the KNP, which could be hotspots for precipitating the occurrence of ASF outbreaks. However, the predisposing factors prior to outbreak occurrence have not been adequately investigated, except to speculate that illegal poaching of warthogs/bush pigs likely contribute in the disease transmission to domestic pigs. The practice of processing hunted warthog/bush pig meat within villages where domestic pigs are reared could facilitate transmission and spread of ASFV in these risk areas.

Molecular analyses of this study seem to intimate on the possible involvement of sylvatic hosts in the epidemiology of ASF in Zambia, especially for viral genotypes I and XIV. For example, phylogenetic analysis of the *p72* gene showed that genotype I ASFVs associated with 2013–2015 outbreaks grouped together with isolates detected from soft ticks in Livingstone (Figure 3). Similarly, genotype XIV viruses detected in domestic pigs in Kalomo (2013) and Kasempa (2014) Districts were closely related to a tick virus (NYA 1/2) isolated in 1986 (Figure 3). Moreover, the virus identified in Kalomo was 100% identical to that of NYA 1/2 in amino acid sequence comparisons of the CVR (Figure 5). In the *p54* phylogeny, genotypes Ie (viruses from ticks) and genotype 1f (2001–2002 viruses from pigs) were closely related (Figure 4a). Also, phylogenetic analysis of the *p30* gene revealed the clustering together of tick-associated viruses LIV 5/40 and LIV 10/11 with that of ZAM 2001/6, which was found in pigs (Figure 4b). Along with epidemiological data, which indicated that the initial outbreaks involving both genotypes I and XIV occurred near the KNP ecosystem, where infected sylvatic hosts were identified previously [13], these findings support the idea of a possible sylvatic role in the epidemiology of ASF in Zambia. However, further studies are needed to better understand the transmission pathways of ASF at the wildlife-livestock interface areas in Zambia. 

Considering that ASFV was readily isolated from *Ornithodoros moubata* ticks in National Parks and GMAs in regions other than the Eastern Province of Zambia where domestic pigs were present [13], it remains enigmatic as to why only this province reported ASF outbreaks prior to 1989. This could be related to differences in pig population and agricultural practices between Eastern Province and other regions of Zambia. Before 1989, Eastern Province had the largest proportion of the indigenous pig population, which was reared under a free-range management system [13]. In other parts of Zambia such as Southern Province, the major agricultural activities are cattle rearing and crop production. Probably, due to very severe outbreaks of Corridor disease in this region, the cattle population was dramatically reduced during the 1990s and this led to increased interest in pig keeping [30]. Large pig populations or an increase in the number of pigs appears to be a risk factor for ASF [6]. It is also possible that ASF outbreaks may have been occurring in very remote areas and thus were not reported. Such outbreaks tend to receive attention when they extend to urban areas probably due to their impact on the commercial pig production sector.

In the study period, there were no reports of ASF outbreaks in Western, Luapula and Muchinga (formerly part of Northern) Provinces (Figure 1). This could partly be explained by the fact that historically, these areas had the lowest pig population densities [13] and trade in pigs and pig products was not a major activity in these regions. However, for Western Province, the areas of concern for possible occurrence of ASF are those bordering the KNP and surrounding GMAs, especially in the southeastern part of the province where pig keeping is presently gaining interest. For Luapula and Muchinga Provinces, the most likely mode of introduction of the disease is through movement of infected pigs and/or pork products from Mbala District, which is currently experiencing frequent ASF outbreaks [16,59]. In fact, as this report was being prepared, outbreaks of ASF were reported for the first time in Chinsali and Isoka Districts of Muchinga Province killing over 1000 pigs [60,61].

Although our findings have revealed that the epidemiology of ASF in Zambia is quite complex and therefore poses a huge challenge for the control of the disease, there are measures that have been applied elsewhere in sub-Saharan Africa, which can be explored and adopted to the local situation. For instance, in countries such as Kenya, South Africa, Zimbabwe, Botswana and Namibia, application of measures aimed at preventing contact between domestic pigs and the sylvatic hosts of ASFV have shown a high level of success in preventing outbreaks [6]. However, in Zambia, there is a need to first provide tangible evidence of the areas where transmission of ASFV from sylvatic hosts to domestic pigs occurs. From the forgoing, we foresee a situation where Southern Province could be a source of ASFVs with potential to cause countrywide or even regional outbreaks. As such, surveillance and monitoring of ASF along with stringent enforcement of movement restrictions of pigs and pork products during outbreaks could help avert significant economic losses associated with this disease.

Some outbreaks documented in this study were linked to Chibolya market in Lusaka District. Therefore, in addition to conducting serological surveillance and monitoring of ASF at slaughter slabs, abattoirs and butcheries, such facilities at high risk of ASF should be identified and placed under strict supervision of veterinary, public health and local government (council) authorities. Indeed, implementation of basic biosecurity measures throughout the pig value chain (from producers to consumers) would go a long way in minimizing outbreaks. Rearing of pigs in residential areas such as compounds (slums or shantytowns) should be strictly prohibited. Trade and movement of pigs and/or their products facilitated ASF spread in most outbreaks. Thus, quarantine and animal movement control must be strictly applied during outbreaks. As some of the outbreaks occurred or appear to have emerged at border areas with neighboring countries (e.g., outbreaks in Kazungula, Livingstone, Solwezi and Mbala Districts), such events pose a risk to the region’s pig industry and thus tightening of porous borders in collaboration with neighboring countries for the control of ASF cannot be overemphasized. Further, community engagement is very important in the control of ASF. The community must be educated on good pig farming practices, basic biosecurity measures and on how to recognize and respond during an ASF outbreak. Indeed, in Côte d’Ivoire where ASF was successfully eradicated in the recent past, awareness campaigns helped to limit dangerous practices that could fuel the spread of ASF by producers [62].

## 6. Conclusions

The present study has clearly demonstrated that other than the 1989 ASF outbreak at Mpima Seminary, which appears to have originated from Eastern Province, the rest of the outbreaks most likely emerged from areas considered to be nonendemic. Therefore, the current ASF control policy in Zambia, which largely focuses on limiting the disease to Eastern Province, may need to be revised as ASFVs capable of causing countrywide havoc on the pig industry could emerge from elsewhere.

## Figures and Tables

**Table 1:** Amino acid sequences of the tetrameric repeats of the CVR of the *B602L* gene identified in Zambia *.

Virus Name	*p72* Genotype	Cumulative CVR Variants Identified	CVR Sequence	No. of Repeats
KAV 89/1	VIII	1	AVSVSVSOVNAVNOVVNVNOVVNVOVOOV	29
ZON 88/1	VIII	2	AVSVSVSVSOVNAVNOVVNVVOVVNVOVOOV	31
CHM 88/1	VIII	3	AVSVSOVNAVNOVVNVOVOOV	21
ZAM/13/Lusaka	I	4	BNAF	4
LIV 13/33	I	5	BNADBNAFTBTDBNAF	16
ZAM 2002/2	I	6	BNAFNBTDBNAF	12
LIV 12/17	I	7	BNAAFNBTAFF	11
LIV 10/11	I	8	BNAAAAAF	8
ZAM/14/Chipata	II	9	BNABNDBTDBNAAG	14
ZAM/13/Mbala	II	10	BNDBNDBNAA	10
ZAM/13/Kalomo	XIV	11	BNWNBVF	7
ZAM/14/Kasempa	XIV	12	AVVWNVWNVWVVF	13
KAB 6/2	XI	13	AVBNOVAVVBNOVAVVOV	18
SUM 14/11	XIII	14	AVNAVSSSSVOVBNASOV	18

* Key: (CAST, CVST, CTST, CASI = A) (CADT, CADI, CTDT, CAGT, CVDT = B) (GAST, GANT = C) (CANT, CAAT = F) (NVDT, NVGT, NVDI = N) (DVDT, NVNT=T) (RAST = H) (SAST = S) (NANI, NADI, NASI = O) (NAGT, NAST, NAVT, NADT, NANT, NANV = V) (CASM = D) (CTNT = G) (NEDT = M) (SADT, SVDT = W) (NIDT, NTDT = U) (NTDI = X) (GTDT = J) (CTSP = K) (YTNT = L).

## Supplementary Materials

The following are available online at www.mdpi.com/1999-4915/9/9/236/s1, Table S1: Summary of African swine fever viruses detected in Zambia used in this study.
