# Supplementary material for: The Epidemiology of African Swine Fever in “Nonendemic” Regions of Zambia (1989–2015): Implications for Disease Prevention and Control
Source: Viruses. 2017 Aug 23;9(9):236. doi: 10.3390/v9090236 (PMC5618003; doi:10.3390/v9090236)
Supplement: Supplementary file 1 [file viruses-09-00236-s001.pdf]

Table S1. Summary of African swine fever viruses detected in Zambia used in this study*

Virus Name	District/area	Year	Host	*p72* GenBank accession no. [Reference]	*p72* Genotype	*p54* GenBank accession no. [Reference]	*p54* Genotype	*p30* GenBank accession no. [Reference]	CVR
ZAM 2001/2	Kafue	2001	Pig	AY494555 [22]	I	KF015915 (Unpublished)	If	JQ764958 (Unpublished)	MF322715 (This study)
ZAM 2001/5	Monze	2001	Pig	AY494558 [22]	I	KF015917 (Unpublished)	If	JQ764961 (Unpublished)	Not done
ZAM 2001/1	Lusaka	2001	Pig	AY494554 [22]	I	Not done	Not done	JQ764957 (Unpublished)	MF322716 (This study)
ZAM 2001/3	Mazabuka	2001	Pig	AY494556 [22]	I	KF015916 (Unpublished)	If	JQ764959 (Unpublished)	Not done
ZAM 2002/1	Lusaka	2002	Pig	AY494559 [22]	I	KF015943 (Unpublished)	If	JQ764962 (Unpublished)	MF322711 (This study)
ZAM 2002/2	Lusaka	2002	Pig	JX403680 (Unpublished)	I	KF736426 (Unpublished)	If	JQ764963 (Unpublished)	MF322710 (This study)
ZAM 2002/3	Lusaka	2002	Pig	JX403681 (Unpublished)	I	Not done	Not done	Not done	Not done
ZAM 2001/4	Namwala	2001	Pig	AY494557 [22]	I	KF736413 (Unpublished)	If	JQ764960 (Unpublished)	MF322714 (This study)
ZAM 2001/6	Lusaka	2001	Pig	Not done	Not done	KF015918 (Unpublished)	If	JX524222 (Unpublished)	MF322713 (This study)
LIV 13/33	Livingstone	1983	Tick	AY494560 [22]	I	KF015898 (Unpublished)	VIIIb	Not done	MF359237 (This study)

Table S1 continued

LIV 9/31	Livinstone	1983	Tick~~s~~	AY351538 [22]	I	KF015928 (Unpublished)	Ie	JQ764966 (Unpublished)	MF322724 (This study)
LIV 12/17	Livingstone	1983	Tick	AY351524 [22]	I	KF015929 (Unpublished)	Ie	JQ764967 (Unpublished)	MF322723 (This study)
LIV 5/4	Livingstone	1983	Tick~~s~~	AY351537 [22]	I	KF015930 (Unpublished)	Ie	Not done	MF322722 (This study)
LIV 5/40	Livingstone	1982	Tick~~s~~	AY351536 [22]	I	KF015931 (Unpublished)	Ie	KC867518 (Unpublished)	MF322721 (This study)
LIV 9/35	Livingstone	1983	Tick~~s~~	AY351539 [22]	I	KF736415 (Unpublished)	Ie	JQ764965 (Unpublished)	Not done
LIV 10/11	Livingstone	1983	Tick~~s~~	AY351535 [22]	I	KF015932 (Unpublished)	Ie	KC867519 (Unpublished)	MF322720 (This study)
ZAM/15/Lusaka	Lusaka	2015	Pig	LC088171 [39]	I	LC088174 [39]	Ie	LC213616 (This study)	LC174776 [16]
ZAM/13/Lusaka	Lusaka	2013	Pig	LC174754 [16]	I	LC174764 [16]	Id	LC213614 (This study)	LC174770 [16]
ZAM/15/Kitwe	Kitwe	2015	Pig	LC088172 [39]	I	LC088175 [39]	Id	LC213617 (This study)	LC174775 [16]
ZAM/15/Solwezi	Solwezi	2015	Pig	LC088173 [39]	I	LC088176 [39]	Id	LC213618 (This study)	LC174774 [16]
ZAM/13/Choma	Choma	2013	Pig	LC174756 [16]	I	LC174759 [16]	Id	LC213610 (This study)	LC174773 [16]
ZAM/13/Chongwe	Chongwe	2013	Pig	LC174757 [16]	I	LC174758 [16]	Id	LC213609 (This study)	LC174772 [16]

Table S1 continued

ZAM/13/Kazungula	Kazungula	2013	Pig	LC174755 [16]	I	LC174763 [16]	Id	LC213613 (This study)	LC174771 [16]
ZAM/14/Chipata	Chipata	2014	Pig	LC174751 [16]	II	LC174760 [16]	IIb	LC213611 (This study)	LC174766 [16]
LUS 93/1	Lusaka	1993	Pig	AY351563 [22]	II	EU874377 (Unpublished)	IIa	EU874275 (Unpublished)	MF322719 (This study)
ZAM/13/Mbala	Mbala 2013	2013	Pig	LC174750 [16]	II	LC174765 [16]	IIa	LC213615 (This study)	LC174769 [16]
PHW 88/1	Chipata	1988	Pig	AY351567 [22]	VIII	EU874366		EU874257	DQ874361 [26]
TMB 89/1	Petauke	1989	Pig	AY351557 [22]	VIII	KF015937 (Unpublished)	VIIIb	JQ764886 (Unpublished)	DQ874370 [26]
TBM 89/1	Petauke	1989	Pig	AY351556 [22]		EU874361 (Unpublished)	VIIIa	EU874285 (Unpublished)	Not done
KLI 88/2	Petauke	1988	Pig	AY351553 [21]	VIII	EU874347 (Unpublished)	VIIIa	EU874258 (Unpublished)	DQ874384 [26]
KAV 89/1	Katete	1989	Pig	AF449470 [21]	VIII	KF015902 (Unpublished)	VIIIb	JQ764890 (Unpublished)	DQ890169 [26]
KAV 89/3	Katete	1989	Pig	JX467635 (Unpublished)	VIII	KF015900 (Unpublished)	VIIIb	KC867508 (Unpublished)	MF359235 (This study)
JON 89/13	Petauke	1989	Pig	AF449469 [21]	VIII	KF015901 (Unpublished)	VIIIa	JQ764889 (Unpublished)	AY538728
KAL 88/1	Chipata	1988	Pig	AF449468 [21]	VIII	KF736412 (Unpublished)	VIIIb	Not done	AY538732
GUL 88/1	Katete	1988	Pig	AY351521 [22]	VIII	Not done	Not done	KC867514 (Unpublished)	DQ874385 [26]
CHM 88/1	Petauke	1988	Pig	AY351520 [22]	VIII	KF015925 (Unpublished)	VIIIb	JQ764884 (Unpublished)	DQ874357 [26]
CHJ 89/1	Petauke	1989	Pig	AY351519 [22]	VIII	Not done	Not done	Not done	DQ874363 [26]
YEL 88/4	Petauke	1988	Pig	AY351558 [22]	VIII	KF015938 (Unpublished)	VIIIb	JQ764887 (Unpublished)	DQ874362 [26]
KANA 89/1	Katete	1989	Pig	AY351523 [22]	VIII	KF736422 (Unpublished)	VIIIb	KF736440 (Unpublished)	DQ874365 [26]

Table S1 continued

CHG 88/1	Katete	1988	Pig	AY351552 [22]	VIII	Not done	Not done	JQ764883 (Unpublished)	DQ874383 [26]
CHK 89/2	Chipata	1989	Pig	AY351526 [22]	VIII	KF015921 (Unpublished)	VIIIb	JQ764882 (Unpublished)	DQ874379 [26]
TEN 89/1	Petauke	1989	Pig	AY351556 [22]	VIII	KF736424 (Unpublished)	VIIIb	KF736442 (Unpublished)	MF322717 (This study)
NKZ 88/1	Petauke	1988	Pig	AY351554 [22]	VIII	KF015934 (Unpublished)	VIIIb	JQ764885 (Unpublished)	DQ874360 [26]
MAN 89/2	Katete	1989	Pig	AY351562 [22]	VIII	KF015940 (Unpublished)	VIIIb	JQ764881 (Unpublished)	Not done
ZAW 88/1				AY351559 [22]	VIII	KF015941 (Unpublished)	VIIIb	JQ764891 (Unpublished)	DQ874378 [26]
ZON 88/1	Katete	1988	Pig	AY351560 [22]	VIII	KF015939 (Unpublished)	VIIIb	JQ764888 (Unpublished)	DQ874388 [26]
MPO 89/1	Petauke	1989	Pig	AY351541 [22]	VIII	KF736418 (Unpublished)	VIIIb	KC867520 (Unpublished)	DQ874368 [26]
MPI 89/1	Kabwe	1989	Pig	AY351540 [22]	VIII	Not done	Not done	Not done	MF359238 (This study)
KAB 6/2	Livingstone	1983	Tick	AY351522 [22]	XI	EU874331 (Unpublished)	XI	EU874289 (Unpublished)	MF359239 (This study)
MFUE 6/1	Mfue (LNP) Park)	1982	Tick	AY351561 [22]	XII	Not done	Not done	KC867522 (Unpublished)	Not done
SUM 14/11	Sumbu NP	1983	Tick	AY351542 [22]	XIII	EU874357 (Unpublished)	XIII	EU874287 (Unpublished)	MF322712 (This study)
ZAM/13/Kalomo	Kalomo	2013	Pig	LC174752 [16]	XIV	LC174761 [16]	XIV	LC213612 (This study)	LC174767 [14]
NYA 1/2	Kalomo	1986	Tick	AY351555 [22]	XIV	EU874330 (Unpublished)	XIV	EU874302 (Unpublished)	MF322718 (This study)
ZAM/14/Kasempa	Kasempa	2014	Pig	LC174753 [16]	XIV	LC174762 [16]	XIV	Not done	LC174768 [16]

*Unpublished sequences were sequenced at Onderstepoort Veterinary Research, Agricultural Research Council, Onderstepoort, South Africa.
